# Replacing a Cysteine Ligand by Selenocysteine in a [NiFe]-Hydrogenase Unlocks Hydrogen Production Activity and Addresses the Role of Concerted Proton-Coupled Electron Transfer in Electrocatalytic Reversibility

**DOI:** 10.1021/jacs.4c03489

**Published:** 2024-05-15

**Authors:** Rhiannon M. Evans, Natalie Krahn, Joshua Weiss, Kylie A. Vincent, Dieter Söll, Fraser A. Armstrong

**Affiliations:** † Department of Chemistry, University of Oxford, Oxford OX1 3TA, United Kingdom; ‡ Department of Biochemistry and Molecular Biology, University of Georgia, Athens, Georgia 30602, United States; § Department of Molecular Biophysics and Biochemistry, Yale University, New Haven, Connecticut 06511, United States; ∥ Department of Chemistry, Yale University, New Haven, Connecticut 06520, United States

## Abstract

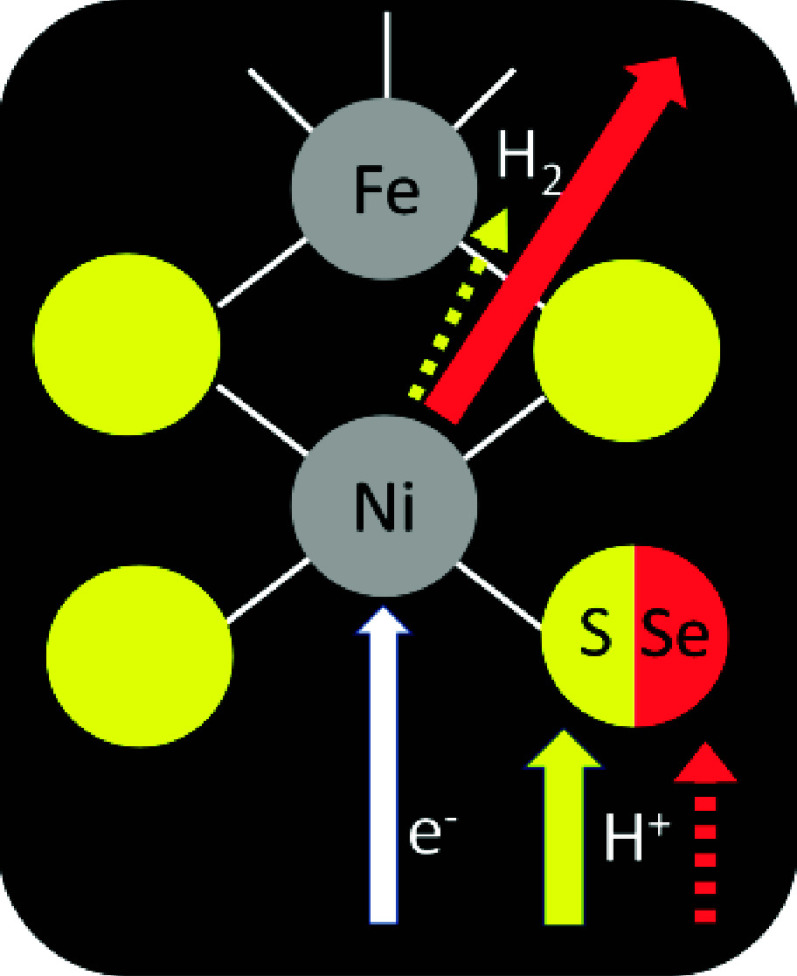

Hydrogenases catalyze hydrogen/proton interconversion that is normally electrochemically reversible (having minimal overpotential requirement), a special property otherwise almost exclusive to platinum metals. The mechanism of [NiFe]-hydrogenases includes a long-range proton-coupled electron-transfer process involving a specific Ni-coordinated cysteine and the carboxylate of a nearby glutamate. A variant in which this cysteine has been exchanged for selenocysteine displays two distinct changes in electrocatalytic properties, as determined by protein film voltammetry. First, proton reduction, even in the presence of H_2_ (a strong product inhibitor), is greatly enhanced relative to H_2_ oxidation: this result parallels a characteristic of natural [NiFeSe]-hydrogenases which are superior H_2_ production catalysts. Second, an inflection (an *S*-shaped “twist” in the trace) appears around the formal potential, the small overpotentials introduced in each direction (oxidation and reduction) signaling a departure from electrocatalytic reversibility. Concerted proton–electron transfer offers a lower energy pathway compared to stepwise transfers. Given the much lower proton affinity of Se compared to that of S, the inflection provides compelling evidence that concerted proton–electron transfer is important in determining why [NiFe]-hydrogenases are reversible electrocatalysts.

Unlike almost all chemical examples, many redox enzymes behave as efficient *reversible* electrocatalysts when attached to an electrode.^1−5^ In electrocatalysis, key parameters are the electron-transfer (ET) steps that determine the electrode potential needed to drive the reaction and nonelectron-transfer steps that determine the limiting current magnitude. Sluggish ET introduces an overpotential barrier beyond that required for a reversible process (where the current responds to a minute departure from the formal potential). For electron-transport enzymes, the catalytic cycle includes transfers of electrons and protons, the former by long-range tunneling^6^ and the latter by short-range hopping assisted by mobile side chains and water molecules.^7,8^ A fundamental requisite for reversible electrocatalysis is the temporal coupling of these transfers to give a concerted proton-coupled electron-transfer (PCET) process.^9−14^ Concerted PCET affords a lower activation barrier compared to stepwise reactions, as summarized by Mayer, Hammes-Schiffer, Hammarström, and co-workers.^15−19^

Briefly, transferring an electron and a proton simultaneously to a buried site in a protein diminishes the Born energy penalty and may avoid an electronically unfavorable intermediate. For a bidirectional cyclic process, the advantage is illustrated with a square scheme (Figure 1A) where the options are to go around the square (sequential) or across (concerted).^12^ The former imposes an overpotential cost in each direction because electron (or proton) transfers alone produce electrostatically or electronically unstable states, with respective steps being associated with reduction potentials *E* or *E*_H_ and protonation constants p*K*_O_ or p*K*_R_.

**Figure 1:**
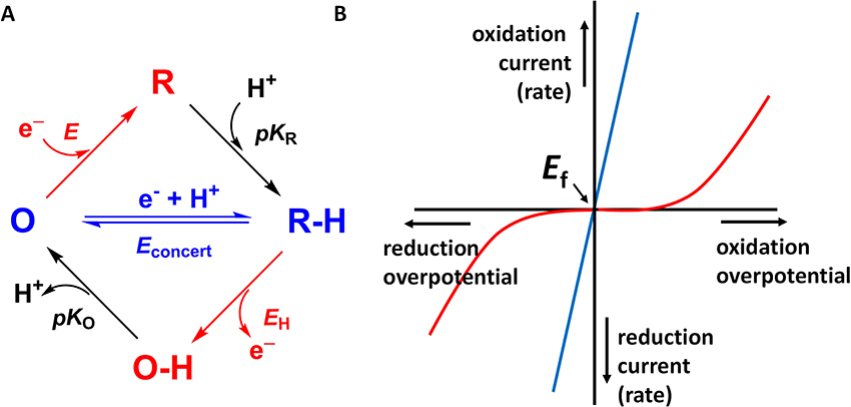
(A) Square scheme showing oxidized species O interconverting with reduced species R–H by either a favorable concerted PCET process (blue) or separate steps involving relatively unstable intermediates due to retarded proton transfer (red followed by black). (B) Voltammograms corresponding to the reversible case resulting from concerted PCET (blue) and the irreversible case resulting from electron transfer preceding proton transfer (red): the latter raises the overpotential requirement in each direction. Formal potential indicated as *E*_f_.

The difference between concerted and sequential PCET is manifested in the catalytic voltammograms, represented in Figure 1B for a bidirectional system with both oxidized and reduced states being present. A concerted process yields a trace (blue) that cuts sharply through the formal potential, whereas sequential transfers produce an inflection (red) reflecting the additional overpotential needed to drive the reaction in each direction.

Hydrogenases are excellent exemplars of reversible electrocatalysis, their inherent activities being comparable with Pt metals.^20,21^ Moreover, they have long been important subjects for protein film electrochemistry (PFE), a suite of techniques providing exquisite, complementary information on redox enzymes.^21−23^ For [FeFe]-hydrogenases, much is now established about the mechanism of H_2_ activation by the active-site H-cluster.^24,25^ Experimentally, the specific role of concerted PCET in electrocatalytic reversibility is difficult to isolate among other contributing factors:^1,26^ however, for two [FeFe]-hydrogenases, mild disruption of a remote proton-transfer pathway by exchanging a Glu for Asp caused the reversible electrocatalytic trace to become sigmoidal (an inflection appearing in the otherwise continuous potential dependence).^27^ Adapting the concept shown in Figure 1, the observations, interpreted as retarded proton mobility, confirmed that *long-range* concerted PCET underpins the reversibility of [FeFe]-hydrogenases.

An interesting case arises for [NiFe]-hydrogenases, where interconversion between H_2_ and H^+^ occurs at a site containing a Ni tetrathiolato (four-cysteine) complex linked via two of the cysteine-S atoms to a Fe^II^(CN)_2_CO fragment (Figure 2A).^28^ During the catalytic cycle summarized in Figure 2B, the Ni atom undergoes changes in the oxidation state (3+, 2+, 1+). There is overwhelming evidence that one or both protons consumed or generated at the active site enter or exit via a pathway comprising nickel-hydrido species, a coordinating (non-bridging) cysteine-S, and the side-chain carboxyl of a glutamate able to approach within H-bonding distance of that specific cysteine S atom.^29−38^ A relay pathway is defined for long-range H^+^ transfer, i.e., solvent ↔ Glu ↔ Cys-S ↔ Ni, as outlined in Figure 2A, based on the structure of Hydrogenase-2 (Hyd-2) from *Escherichia coli*.^39^ The electron is transferred to the proximal [4Fe–4S] cluster located approximately 11 Å from the Ni atom.

**Figure 2:**
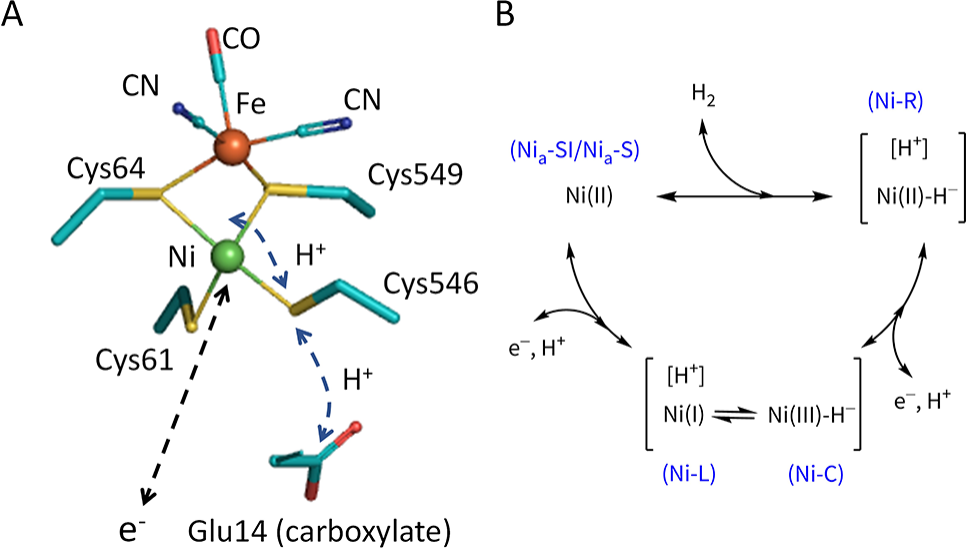
(A) Active site of a [NiFe]-hydrogenase, showing the pathway proposed for H^+^ transfer between its coordination site (as a hydrido ligand) on the Ni atom (Ni–C) via an inner-shell cysteine-S to an outer-shell glutamate and ultimately to solvent. Residues are numbered according to the sequence of *E. coli* Hyd-2. (B) Catalytic cycle showing the oxidation states of intermediates Ni–R, Ni–C, Ni–L, and Ni_a_–SI (Ni_active_–EPR silent).

Our attention focuses on the lower stages of the catalytic cycle involving long-range electron–proton transfer (the upper horizontal process is concerned with H–H bond formation/cleavage and the elusive interaction with the H_2_ molecule).^40,41^ The central (and best characterized) intermediate is a Ni(III)-hydrido species known as Ni–C (isoelectronic with Ni(I)–H^+^ which corresponds to the R–H species in Figure 1A). The Ni–C state is in tautomeric equilibrium with Ni(I) species known collectively as Ni–L: IR spectroscopic studies have revealed that both Ni–L and Ni–R exist in several forms, differing in the location of the H^+^ that has migrated locally without leaving the enzyme.^32,33,35,38^ The Ni–L species form upon illumination at low temperature, but their detection under normal catalytic conditions has largely been restricted to O_2_-tolerant [NiFe] hydrogenases, suggesting that the equilibrium otherwise strongly favors Ni–C.^33,38,42,43^ During H_2_ oxidation, Ni–C is converted to a Ni(II) form known as Ni_a_–SI. Investigations of the Ni–C to Ni_a_–SI interconversion by transient spectroscopy lend strong support for a concerted process,^31,32^ and recent studies of the regulatory hydrogenase from *Cupriavidus necator* by cryo-IR and EPR produced a particularly detailed picture of the proton-transfer steps in that enzyme, elaborating on the pathway shown in Figure 2A.^38^

The crucial cysteine both coordinates Ni and serves as a H^+^ mediator. An attractive approach for probing the role of a cysteine-S atom is to exchange the cysteine for a selenocysteine (Sec, one-letter code U) by recombinant methods.^44−46^ Selenium is only slightly larger than sulfur, and has a similar electronegativity, but its proton affinity is much lower; this property is passed on to Sec, for which the p*K*_a_ of the free amino acid is 5.2 compared to 8.3 for Cys.^47−52^ In a notable subclass of [NiFe]-hydrogenases, the same cysteine is replaced by selenocysteine: compared to standard [NiFe]-hydrogenases, [NiFeSe]-hydrogenases have higher activity for proton reduction with very little product (H_2_) inhibition.^53−56^ We recently used PFE to investigate the consequences of replacing each of the four Cys coordinating the Ni, by Sec, in the O_2_-tolerant Hydrogenase-1 (Hyd-1) from *E. coli*.^57^ Despite identifying aspects underlying O_2_ tolerance, we could not address the role of each Sec in catalytic proton transfer because, under neutral pH conditions, Hyd-1 is not a bidirectional catalyst: it catalyzes only irreversible H_2_ oxidation.^58^ We have now made the corresponding Cys-to-Sec exchanges with the counterpart standard hydrogenase of *E. coli*, Hyd-2: unlike Hyd-1, Hyd-2 is a reversible electrocatalyst of the 2H^+^/H_2_ reaction, albeit with H^+^ reduction activity that is strongly inhibited by H_2_.^39^ We could thus determine how each Cys contributes to the *bidirectional* electrocatalytic “signature”, interest being focused on Cys-546, implicated in concerted PCET and replaced by Sec in [NiFeSe]-hydrogenases.

To produce Hyd-2 Sec variants, we followed the gene-expression protocol used previously to overproduce native Hyd-2^59^ combined with the technology for site-specific Sec insertion.^45^ Details are given in the Supporting Information. Hyd-2 was produced from *E. coli* with a chromosomally encoded C-to-U mutant *hyb*C gene and a plasmid encoded, C-terminally hexa-His-tagged *hyb*O gene (pOC)^59^ (Figure S1). The Cys codons at positions 61, 64, 546, and 549 in hybC were individually mutated to TAG to create four new *E. coli* strains (Table S1). Transforming these strains with pOC^59^ and pSecUAG-Evol2^45^ plasmids yielded final expression strains. Mass spectrometry confirmed maturely processed enzyme with the C-terminal “assembly peptide”^60^ missing and Sec insertion at the expected TAG positions (Figure S1). All four variants were active in steady-state H_2_ oxidation assays, although at lower levels (per mg enzyme) than measured for native Hyd-2 (Table S2).

Despite low yields, definitive observations were made using PFE which requires only minute enzyme quantities and focuses on electrocatalytic signature (reversibility, catalytic bias) rather than absolute activity.^21−23^ Catalytic cyclic voltammograms were recorded for native Hyd-2 and each variant, adsorbed at a pyrolytic graphite “edge” (PGE) electrode (Supporting Information). The results are displayed in Figure 3 alongside the corresponding Cys/Sec exchange position. The voltammetry for the C546U variant is markedly different. Under 100% H_2_, the current due to H^+^ reduction at −0.2 V vs RHE is larger than that for H_2_ oxidation at +0.2 V vs RHE: catalytic bias has thus been erased. The comparative result after displacing H_2_ with Ar shows that H_2_ is no longer an inhibitor. Close inspection shows that the sharp intersection across the formal 2H^+^/H_2_ potential, expected for reversible electrocatalysis and clearly apparent for native Hyd-2 when lower, less inhibitory H_2_ levels are used (Figure S2),^39,41^ is replaced by a subtle inflection. Figure 4 shows that the inflection persists between pH 5 and 8; activity at pH 9 is too low to distinguish the shape. Once most H_2_ has been replaced by Ar, an inflection is neither expected nor evident, with the greater potential dependence of the H^+^ reduction rate being the only observable.

**Figure 3:**
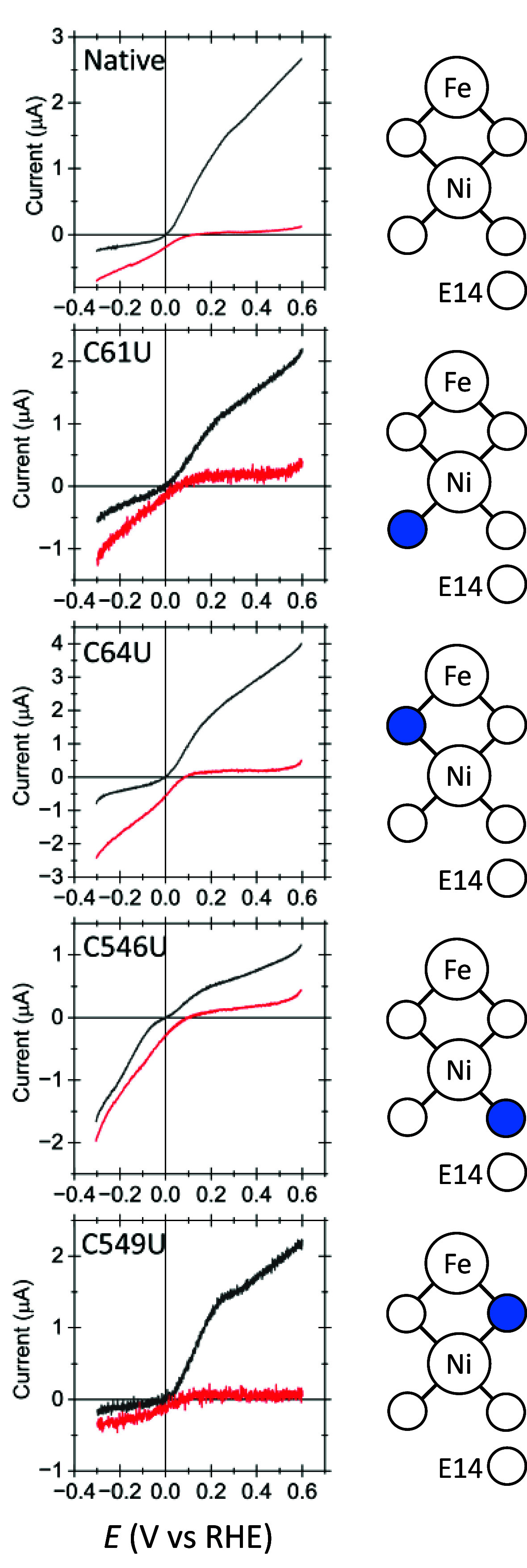
Steady-state catalytic voltammograms recorded for Sec variants under 100% H_2_ (black) and then after replacing the headspace with Ar (red), scan rate 0.5 mVs^–1^. Other conditions: temperature 37 °C; pH = 6.0; electrode rotation rate 1000 rpm. Potential axis has been adjusted to approximate to the reversible hydrogen electrode (RHE) scale.

**Figure 4:**
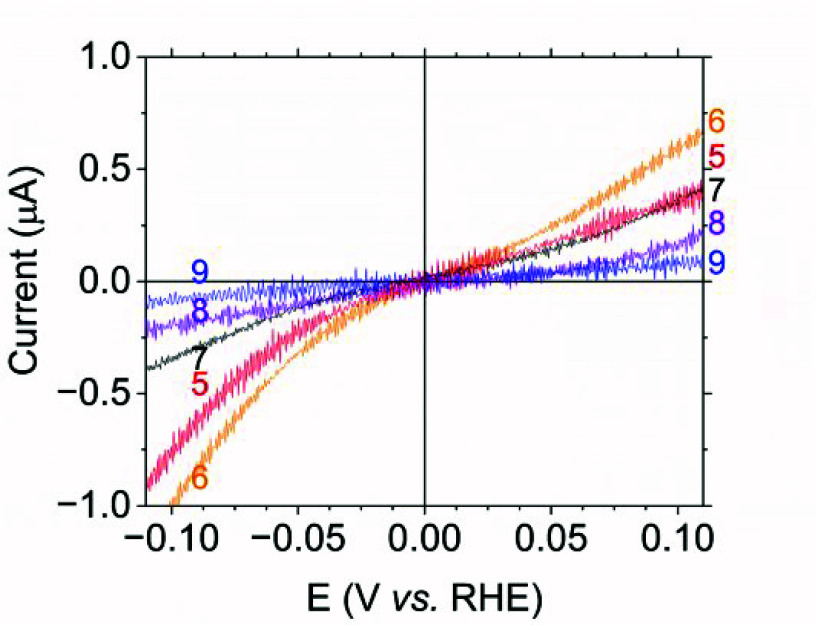
Expanded view showing the catalytic voltammograms for 2H^+^/H_2_ interconversion by the C546U variant over a range of pH values, at 100% H_2_, in the reversible region spanning the formal potential. Scan rate 2 mV s^–1^; temperature 37 °C; electrode rotation rate 1000 rpm.

The C546U exchange thus renders Hyd-2 fully bidirectional but with a small but discernible decrease in reversibility. Although substitution of Cys for Sec at this Ni-coordinating site might render electron transfer more sluggish through intractable electronic or structural effects, a more plausible explanation is that electron and proton transfers, occurring in concert (at least for Ni–C to Ni_a_–SI interconversion),^31,32^ have become temporally separated. Aside from inevitable alterations in dynamic local structure, the net impact of which is difficult to predict, the obvious factor is the lower (thermodynamic) proton affinity of Se compared to S, which may override the greater nucleophilicity (kinetic) advantage expected for the former:^48,50,52^ compared to thiolate, a selenide base is less able to stabilize H^+^ in transit. No inflection is observed in PFE studies of natural [NiFeSe]-hydrogenases,^53−55^ suggesting other factors ensure that Se imposes no H^+^ transfer penalty for those enzymes.

Decreased product (H_2_) inhibition is one reason natural [NiFeSe]-hydrogenases have higher H_2_ production activities.^53−55^ Replacement of S by Se may influence the elusive interaction between Ni and molecular H_2_ or modify the activity of Ni–R states (one of which may be protonated at C546).^34^ For O_2_-tolerant Hyd-1, the equivalent position was identified to help confer O_2_ tolerance.^57^

Although at a qualitative stage, the new data establish that long-standing mechanistic hypotheses surrounding [NiFe]- and [NiFeSe]-hydrogenases will be amenable to closer examination once larger quantities of variants can be produced. The subtle inflection introduced by a specific mutation highlights the influence of concerted proton–electron transfer in minimizing overpotential and achieving reversible electrocatalysis that is so rare in electrochemical research. Even so, we note that it is possible to account for reversible electrocatalysis without explicit consideration of concerted PCET.^26^ Finally, with early organisms having a limited thermodynamic range available for electron-transport chains, enzyme structures may have evolved to promote concerted PCET for optimizing efficiency in energy processing. By further refining thermodynamic fitness,^61^*overpotential*, a term otherwise used exclusively by electrochemists, would have been an underlying evolutionary driver.^3^

## Supporting Information Available

Plasmid preparation and construction of expression strains, production and characterization of variants, enzyme evaluation, steady-state H_2_ oxidation activity assays, and electrochemical setup; Table S1: Genotypes of strains created and used in this work; Table S2: Steady-state H_2_ oxidation activity assays; Figure S1: Hyd-2 active site highlighting the four Cys residues targeted for Sec insertion, SDS-PAGE showing purification of both the large subunit (HybC) and the small subunit (HybO) of Hyd-2 for each of the four Sec variants, and tandem MS data for all four Sec variants; Figure S2: Steady-state catalytic cyclic voltammograms recorded for native Hyd-2 under increasing levels of H_2_ (PDF)
