# Supplementary material for: Replacing a Cysteine Ligand by Selenocysteine in a [NiFe]-Hydrogenase Unlocks Hydrogen Production Activity and Addresses the Role of Concerted Proton-Coupled Electron Transfer in Electrocatalytic Reversibility
Source: J Am Chem Soc. 2024 May 15;146(25):16971–6. doi: 10.1021/jacs.4c03489 (PMC11212049; doi:10.1021/jacs.4c03489)
Supplement: Supplementary file 1 — ja4c03489_si_001.pdf [file ja4c03489_si_001.pdf]

Replacing a cysteine ligand by selenocysteine in a [NiFe]-hydrogenase unlocks hydrogen production activity and addresses the role of concerted proton-coupled electron transfer in electrocatalytic reversibility

Rhiannon M. Evans,[1] Natalie Krahn,[2] Joshua Weiss,[2] Kylie A. Vincent,[1] Dieter Söll[3,4] and Fraser A. Armstrong[1]

[1]Department of Chemistry, University of Oxford, South Parks Road, Oxford, UK, OX1 3QR; [2]Department of Biochemistry and Molecular Biology, University of Georgia, Athens, GA 30602, [3]Department of Molecular Biophysics and Biochemistry, Yale University, New Haven, CT 06511, USA; [4]Department of Chemistry, Yale University, New Haven, CT 06520, USA

SUPPORTING INFORMATION

Materials and Methods

Plasmid preparation and construction of expression strains

To prepare HJ001-hyp strains for Sec insertion, the natural Sec insertion machinery (*selABC*) was removed via recombination[1] to produce NKHJ. Following this removal, C to U mutations were required on the chromosomal copy of *hybC*. Native *hybC* in a pUC18 plasmid (Addgene; plasmid 50004) was used as a template for mutation of the Cys positions (61, 64, 546, and 549) to TAG using the QuikChange II Site-Directed Mutagenesis protocol (Agilent Technologies). Flanking regions (50 bp) homologous to the surrounding *hybC* gene sequence in the NKHJ strain were added to both ends of the successfully mutated *hybC* gene in pUC18. Primers were acquired from Keck Biotechnology Resource and DNA sequencing was performed at the Keck DNA Sequencing Facility at Yale University. The resulting TAG variants of *hybC* were used to replace the wild-type *hybC* gene in the genome of NKHJ cells via recombination[1] to generate strains NK168 to NK171 (Table S1).

Production and characterization of variants

NK strains were transformed with pOC (pQE-80L containing *hybO*) and pSecUAG-Evol2 (Addgene; plasmid 163418) for expression of Sec-containing Hyd-2 variants.[2] Overnight cultures (10 mL) of Luria Broth (LB) containing antibiotics were grown aerobically at 37 °C and then transferred into 6 L LB media with 0.5% (v/v) glycerol, 25 mM sodium fumarate, 0.1% (w/v) arabinose, 10 μM sodium selenite, 100 μg/mL ampicillin and 50 μg/mL kanamycin. Variants were grown anaerobically until OD_{600}=0.4 and then induced with 1 mM isopropyl β-D-1-thiogalactopyranoside for overnight anaerobic expression. All strains were harvested and

membranes solubilized for Hyd-2 purification by Ni-affinity chromatography as previously described.[2]

Enzyme evaluation

All variant enzymes were run on a polyacrylamide gel, and bands corresponding to the large subunit (~ 70 kDa) were cut out for mass spectroscopy analysis. The gel bands were sliced into small pieces and then rinsed twice with 50% acetonitrile/20 mM Triethylamine bicarbonate [diluted with 1M Triethylammonium bicarbonate buffer (Sigma-Aldrich)]. Proteins were reduced and alkylated by dithiothreitol and iodoacetamide, respectively, followed by two more rinses with 50% acetonitrile/20 mM ammonium bicarbonate and dehydrated with 100% acetonitrile and heat. Trypsin digest was performed on the proteins in 20 mM ammonium bicarbonate and the resulting peptides extracted from the gel pieces.

The LC/MS analyses were performed on a Thermo-Fisher LTQ Orbitrap Elite Mass Spectrometer coupled with a Proxeon Easy NanoLC system located at the Proteomics and Mass Spectrometry Facility, University of Georgia. Peptides were resuspended in 2% (v/v) acetonitrile/0.1% (v/v) formic acid and loaded onto a reversed-phase column (self-packed column/emitter with Dr. Maisch ReproSil-pur C18AQ 120 Å, 3 µM resin), then directly eluted into the mass spectrometer. Briefly, the two-buffer gradient elution (0.1% formic acid as buffer A and 99.9% acetonitrile with 0.1% formic acid as buffer B) starts with 0% B, holds at 0% B for 2 minutes, then increases to 40% B over 40 minutes and to 50% B over 15 minutes before ending at 95% B in 5 minutes. The data-dependent acquisition (DDA) method was used to acquire MS data. A survey MS scan was acquired first, and then the top 12 ions in the MS scan were selected for following CID and HCD MS/MS analysis. Both MS and MS/MS scans were acquired by Orbitrap at the resolutions of 120,000 and 15,000, respectively. Data were acquired using Xcalibur software (version 3.0, Thermo Fisher Scientific). Protein identification and modification characterization were performed using Thermo Proteome Discoverer (version 3.0) with Mascot (Matrix Science) and *Escherichia coli* database.

Table S1. Genotypes of strains created and used in this work.

**Strain**	**Genotype**	**Reference**
HJ001-hyp	*IC011 ΔiscR ΔtatD::hypA1-X*	Ref. 2
NKHJ	*HJ001-hyp ΔselABC*	This work
NK168	*NKHJ hybC::hybC61TAG*	This work
NK169	*NKHJ hybC::hybC64TAG*	This work
NK170	*NKHJ hybC::hybC546TAG*	This work
NK171	*NKHJ hybC::hybC549TAG*	This work

Steady-state H_2 oxidation activity assays

The steady-state H_2 oxidation activity of each of the five Hyd-2 enzymes presented in this study was measured by monitoring the change in absorbance of benzyl viologen over time at 604 nm (Table S2). A solution of 2 mM benzyl viologen in 50 mM potassium phosphate buffer,

pH 6, 25 °C was prepared, covered in foil to protect from ambient light, then taken into an anaerobic glovebox (Belle Technologies, $O_2 < 10$ ppm) and allowed to equilibrate with the box N_2 atmosphere overnight. As-isolated enzyme samples were prepared as initial stocks at a concentration of 0.2 mg/mL. In order to measure linear responses, these stocks were then diluted 25-100-fold depending on the activity of each enzyme, and adjusted if required. The activity assay was then performed as previously described.[3] The assay solution was saturated with H_2 sparging for at least 30 mins, and then 1 mL was transferred to a cuvette and the background change in absorbance monitored as a negative control (the cuvette was under constant H_2 flow). The activity assay was initiated by injection of 5 μL of the diluted enzyme sample. Since enzymes were not pre-activated, a lag phase was noted, during which the enzyme is activated and/or there is reduction and oxidation (by trace O_2 in enzyme samples) of benzyl viologen occurring at the same rate. The gradient of the change in absorbance with time was measured at various time points and the steepest gradient used to calculate the turnover rates using an extinction coefficient of 8.4 mM^{-1} cm^{-1} at 604 nm for benzyl viologen.[4]

Table S2. Steady-state H_2 oxidation activity assays for each Hyd-2 enzyme in this study. Errors are reported as the standard error of the mean of at least three repeats. Data collected at 25 °C, pH 6.0, 100% H_2.

**Enzyme**	**H_2 oxidation rate (s^{-1})**	**H_2 oxidation rate (μmol H_2 min^{-1} mg^{-1})**	**Relative activity (%)**
Native Hyd-2	385.4 +/- 30.3	239.0 +/- 18.8	100
61-TAG	8.9 +/- 2.2	5.5 +/- 1.4	2.3
64-TAG	17.0 +/- 1.7	10.5 +/- 1.1	4.4
546-TAG	5.1 +/- 1.9	3.2 +/- 1.2	1.3
549-TAG	7.6 +/- 0.7	4.7 +/- 0.4	2.0

Electrochemical set-up

Electrochemistry was performed in an all-glass electrochemical cell as previously described.[5] The cell was water-jacketed to provide temperature control. The working electrode (WE) comprised a rotating (1000-3000 rpm) pyrolytic graphite 'edge' plane electrode (PGE, 3-9 mm^2), the reference electrode (RE) was a saturated calomel electrode housed in a non-water-jacketed side arm and electrochemically connected to the electrochemical cell via a luggin capillary filled with 0.1 M NaCl. The counter electrode (CE) was a platinum wire. Electrode potentials were referenced first to the Standard Hydrogen Electrode (SHE) using $E_{SHE} = E_{SCE} + 0.241$ V at 25 °C. Since it was important to compare the data at different pH values, the Reversible Hydrogen Electrode scale was adopted for both Figures 3 and 4, using the conversion $E_{RHE} = E_{SHE} - (RT/nF)\ln[H^+]$. Electrochemical measurements were carried out via an Autolab PGSTAT128N utilising Nova 1.10 software (EcoChemie). Protein films were made by first abrading the PGE with sand paper (P400, 3M Hoookit) then rinsing in ultra-pure water

(MilliQ 18.2 MΩ.cm) before applying the protein solution (0.02-0.2 mg/mL) to the graphite surface via pipette, removing and reapplying for approx. 1 minute. The electrode surface was then rinsed in ultrapure water and placed immediately in the electrochemical cell. To allow more easily for comparisons between shapes of cyclic voltammograms without the need for normalisation of currents, the electrode surface was wiped with tissue or cotton wool to lower the electrode coverage and achieve similar H_2 oxidation currents for the different enzymes across different experiments. All experiments were performed in a mixed buffer system comprising MES, HEPES, TAPS, CHES, and NaOAc (at 15 mM each; Melford or Sigma Aldrich) and NaCl (100 mM; Sigma-Aldrich) titrated to the desired pH at the experimental temperature of 37 °C. All enzyme films were reductively activated at pH 6.0 in 100% H_2 prior to each experiment by poising the electrode at -0.659 V and periodically returning to -0.1 V to check for increase in H_2 oxidation current. The gases used (BOC) were of highest purity (>99.99% H_2 and 99.998% Ar) and were controlled via mass flow controllers (Sierra).

Following activation, cyclic voltammograms were performed at pH 6.0 under 100% H_2 or 100% Ar at 1000 scc/min (Figure 3). The potential was raised from -0.659 V at a scan rate 0.5 mV/s. The potential continued to rise until the upper vertex of +0.241 V was reached and then the potential was lowered back down to -0.659 V. The data recorded for the forwards and backwards scans have been averaged to clarify the scan shape in the absence of capacitive current and have been corrected for slight current offsets (5-100 nA).

To assess the pH dependence of the C546U variant, cyclic voltammograms were performed at pH 5.0 - 9.0 under 100% H_2 at 1000 scc/min and 37 °C (Figure 4). The potential was raised from -0.659 V at a scan rate of 2 mV/s. The potential continued to rise until the upper vertex of +0.241 V was reached and then the potential was lowered back down to -0.659 V. The data recorded for the forwards and backwards scans have been averaged to clarify the scan shape in the absence of capacitive current and have been corrected for slight current offsets (5-100 nA). There was significant film instability at pH extremes and so the data presented here were recorded using several films, always measuring at pH 6.0 for each film as a control, i.e. for each pH measured, a cyclic voltammogram was performed at pH 6.0 under otherwise the same experimental conditions either immediately before or immediately after the cyclic voltammogram at the different pH, to ensure the same electrochemical profile was evident and to enable assessment of film instability by comparing the before and after profiles, currents and zero-current crossing point vs RHE. For C546U the inflection about the formal potential was always evident during activation prior to any other experiments being performed.

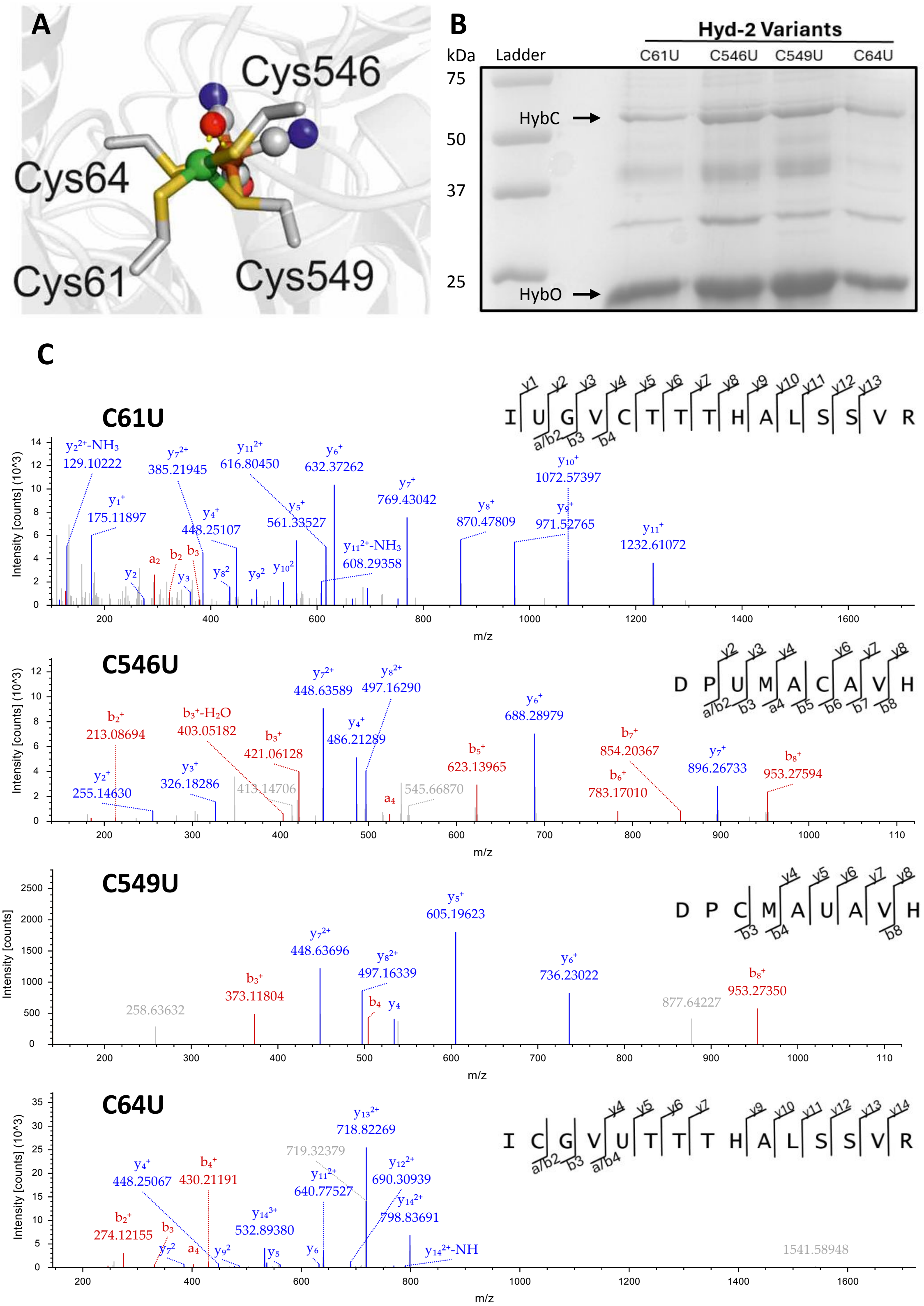

Figure S1. **A**) Hyd-2 active site highlighting the four Cys residues targeted for Sec insertion. **B**) SDS-PAGE shows purification of both the large subunit (HybC) and the small subunit (HybO) of Hyd-2 for each of the four Sec variants. **C**) Tandem MS data for all four Sec variants confirming the presence of Sec at the desired position. No Ser peptides were observed.

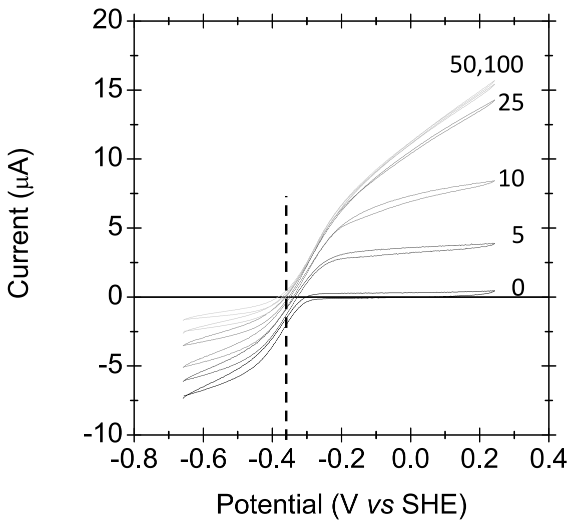

Figure S2 Steady-state catalytic cyclic voltammograms recorded for native Hyd-2 under increasing levels of H_2 (shown as % in Ar). Experimental conditions: 30 °C, pH 6.0, scan rate 1 mV^{-1}, electrode rotation rate 5000 rpm. The formal potential of the H^+/H_2 couple under 100 % H_2 is marked by the dashed line. As expected, under 100 % Ar, the onset of H^+ reduction appears very positive of the formal potential. Increases in H_2 level result in large decreases in H^+ reduction current, while the curve crosses the zero-current line without an inflection. Data originally published as Supplementary Material in Ref. 6, reproduced with permission, American Society for Biochemistry and Molecular Biology.[6]

References

1 Li, X. T., Thomason, L. C., Sawitzke, J. A., Costantino, N. & Court, D. L. Positive and negative selection using the tetA-sacB cassette: recombineering and P1 transduction in Escherichia coli. *Nucleic Acids Res* **41**, e204 (2013). https://doi.org/10.1093/nar/gkt1075

2 Beaton, S. E. *et al.* The structure of hydrogenase-2 from *Escherichia coli*: implications for H(2)-driven proton pumping. *Biochem J* **475**, 1353-1370 (2018). https://doi.org/10.1042/BCJ20180053

3 Evans, R. M. *et al.* Selective cysteine-to-selenocysteine changes in a [NiFe]-hydrogenase confirm a special position for catalysis and oxygen tolerance. *Proc Natl Acad Sci U S A* **118** (2021). https://doi.org/10.1073/pnas.2100921118

4 Cammack, R., Fernandez, V. M. & Hatchikian, E. C. Nickel-Iron Hydrogenase. *Method Enzymol* **243**, 43-68 (1994).

5 Evans, R. M. & Armstrong, F. A. Electrochemistry of Metalloproteins: Protein Film Electrochemistry for the Study of [NiFe]-Hydrogenase-1. *Methods Mol Biol* **1122**, 73-94 (2014). https://doi.org/10.1007/978-1-62703-794-5_6

6 Lukey, M. J. *et al.* How *Escherichia coli* is equipped to oxidize hydrogen under different redox conditions. *J Biol Chem* **285**, 3928-3938 (2010). https://doi.org/10.1074/jbc.M109.067751
